# External handheld loads affect scapular elevation and upward rotation during shoulder elevation tasks

**DOI:** 10.1080/23335432.2024.2332212

**Published:** 2024-03-19

**Authors:** Alan Eldridge, Everett Lohman, Skulpan Asavasopon, Lida Gharibvand, Lori Michener

**Affiliations:** a Physical Therapy Department, Loma Linda University, Loma Linda, CA, US; b Division of Biokinesiology & Physical Therapy, University of Southern California, Los Angeles, CA, US

**Keywords:** Scapula, kinematics, external load, Biomechanics, shoulder, rehabilitation

## Abstract

Altered scapular kinematics is associated with shoulder pain. Resistance exercise is a common treatment; however, the effects of lifting an external load on scapular kinematics is limited. Understanding whether an external handheld load affects scapular kinematics in a healthy population can provide normal values utilized for comparison to individuals with shoulder pain. Currently, no studies have examined the effect of incrementally increased handheld loads. We defined the effects of varying external handheld loads on scapular kinematics during a shoulder elevation task. Healthy participants (*n* = 50) elevated their shoulder in the scapular plane over 4 trials. One trial of no loading (control) and 3 trials with incrementally increased external handheld loads. Scapular kinematic rotations and translations were measured during ascent and descent phases using 3D motion capture. Compared to no load, the highest external load during ascent increased scapular elevation [mean difference = 3.2 degrees (95%CI: 0.9, 5.4), *p* = 0.006], and during descent increased scapular elevation [mean difference = 3.9 degrees (95%CI: 2.8, 5.1), *p* < 0.001] and increased **scapular upward rotation** [mean difference = 4.5 degrees (95%CI: 2.4, 6.6), *p* < 0.001]. External handheld loads result in small **increases in scapular elevation and scapular upward rotation**. These results should be utilized as normal values to compare to individuals with shoulder pain.

## Introduction

Individuals with subacromial pain (SAP), also known as rotator cuff (RC) tendon pathology have altered scapular kinematics compared to healthy controls (Lukasiewicz et al. 1999; Ludewig and Cook 2000; Borstad and Ludewig 2002; Finley et al. 2005; Mell et al. 2005; McClure et al. 2006; Jj et al. 2011; Lawrence et al. 2014; Lopes et al. 2015; Turgut et al. 2016). Mechanistically, deficits in scapular motion or coordination between the scapula and humerus during arm elevation may contribute to SAP via tendon compression or increased tendon loading (Mell et al. 2005; Ludewig and Reynolds 2009). Resistance overhead lifting exercises and functional activities often pose a common source of excessive load, frequently leading to pain; however, resistance exercise is also a commonly recommended evidence-based treatment for SAP to address associated scapular deficits. Unfortunately, not all patients respond favorably to resistance exercise, with approximately 40% experiencing a delayed or no positive benefit (Failla et al. 2022). Understanding how an external handheld load affects scapular kinematics can inform the delivery of resistance exercises to maximize a favorable response. Specifically, this study informs how handheld loading during arm elevation impacts scapular kinematics.

Decreased scapular upward rotation, external rotation, and posterior tilt reduces the subacromial space which may place a compressive stress on the rotator cuff (Seitz et al. 2012a, 2012b; Camargo and Neumann 2019). Altered scapular kinematics may also impact glenohumeral coordinated motion, which can increase load to the RC tendons (Laudner et al. 2006). Excessive scapular elevation in those with RC pathology suggest scapular elevation is associated with excessive tendon loading (Mell et al. 2005). Excessive scapular elevation may contribute to posterior superior tendon impingement between the glenoid and the humerus (Apreleva et al. 2000; Laudner et al. 2006). Excessive scapular elevation and decreased scapular upward rotation are two common alterations in the SAP population (Timmons et al. 2012), however these findings are not consistent across studies as McClure et al. found increased upward rotation in the SAP population (McClure et al. 2006). Identifying whether an external load alters scapular kinematics will allow for optimal load prescription for individuals participating in a strength program, and will provide normal values to compare against when assessing scapular kinematics in a painful population.

Evidence of the effect of load on scapular kinematics is varied, which may be attributed to the magnitude of the load, the plane of humeral elevation, and methods of scapular kinematic assessment across studies. Michiels and de Castro (Michiels and Grevenstein 1995) found no changes in scapular kinematics with external loads, however the load used was low load (2 kg, 5% body weight). Other studies have identified changes in scapular kinematics with external loads, though the findings across studies are varied. Forte et al. (2009) and Wochatz et al. (2021) found increased scapular upward rotation with external loads, while Kon et al. (2008) and Camci et al. (2013) found decreased upward rotation. Findings likely vary due to the variability in load amount utilized across studies. It is possible that the studies that found little or no change in kinematics utilized an insufficient load. A study investigating the effect of incrementally increased and higher loads is needed to enable the delivery of resistance exercise.

The aim of this study is twofold: First, to compare the scapular kinematics during a shoulder elevation task with an external handheld load and a control condition of no external handheld load. Second, to investigate the effects of incrementally increased loads on scapular kinematics. The purpose of the second aim is to attempt to identify the lowest external load that results in altered scapular kinematics. The impact of loading on scapular kinematics will inform the prescription of resistance (loaded) exercise via providing normal values for comparison. We hypothesized that handheld external loads would increase scapular elevation and decrease scapular upward rotation during a shoulder elevation task, and this effect will be greater with higher external loads.

## Methods

Fifty (*N* = 50) healthy participants volunteered for this study (mean age = 30.4 ± 8.5 years). Inclusion criteria was age between 18 and 65. Participants were excluded if they had any current neck or shoulder pain, a history of neck or shoulder surgery, a diagnosis of a chronic pain condition (e.g. fibromyalgia, lupus, rheumatoid arthritis, etc.), was currently taking pain medication, was pregnant, or lacked a minimum of 160 degrees of shoulder elevation. The Loma Linda University Institutional Review Board approved all procedures (Loma Linda, CA, USA). The IRB approval number for this study is 5,190,423. All participants read and signed an informed consent prior to participation.

## Procedures

Prior to the shoulder elevation trials, participants were assessed to determine the maximal load during a peak power output measured during isotonic shoulder elevation in the scapular plane via a 1 repetition maximum (RM) protocol. The results from the 1 RM test were then utilized to individualize the external load during trials 2–4.

### Shoulder Elevation Power Testing Assessment

Using the Baltimore Therapeutic Equipment (BTE) PrimusRS (BTE Hanover, MD), shoulder elevation was performed in the scapular plane (40 degrees from the frontal plane). A PVC wall was created to ensure the participant elevated their arm consistently within the scapular plane. BTE was set to isotonic mode during testing. The participant was instructed to elevate a small hand-held ball which was attached to the BTE via a pulley cable system. Instructions to the participant included: ‘Elevate the ball as high as you can and as fast you can’. Five pounds (2.3 kg) was initially placed on the pulley system for the participant’s first trial of elevation. The participants power output was calculated for each trial of shoulder elevation that they performed. Small increments of resistance were programmed into the BTE system until the participant failed an arm elevation trial. The increments were .45 kgs. to 1.3 kgs. based on how challenging the participant rated the trial. Easy resulted in a 1.3 kg. increase, moderate resulted in a .91 kg. increase, and difficult resulted in a .45 kg. increase. The BTE Primus RS alerted investigators if a participant did elevate their shoulder to the end range of motion. If this occurred, the participant would re-attempt the trial. Failure was defined as a power output that was less than the prior trial. The drop in power output, despite the ability to still perform the repetition through the full ROM, occurs due to form failure per the BTE Primus RS user’s manual. While a participant could complete the shoulder elevation repetition, they are unable to do so with proper form resulting in a lower power score. When subjects lift at 125% of 1 RM they are able to complete the repetition, but with a compensatory strategy. Participants were given 30-s rest between trials to minimize the effects of fatigue. Peak weight utilized (mean 12.34 Kg) and number of trials (mean 5 trials, range 3–8 trials) required were calculated for each participant. The final weight amount was utilized to calculate the participant’s 1 RM that would be lifted during the subsequent shoulder elevation trials. The shoulder elevation power test is similar to a shoulder lifting test, which has been reported as reliable (ICC 0.83–0.96) (Palmer and Uhl 2011).

### Scapular Kinematic Assessment

Kinematics of the scapula and humerus were tracked across the 4 trials in scapular plane humeral elevation (40 degrees anterior to the frontal plane). Participants utilized dumbbells to elevate within the scapular plane. The participant was asked to elevate their arm in the scapular plane, using the specially constructed polyvinyl chloride PVC piping wall as a guide to maintain the scapular plane. To ensure that an adequate load was utilized to potentially achieve kinematic changes, participants lifted at both 100% and 125% of their 1 RM. To assess whether there were any differences between these higher loads and a lower load, 50% of the participants 1 RM was also obtained. Trial 1 was the control condition in which the participant elevated their arm without weight to maximum elevation in the scapular plane. Trial 2 was performed with a handheld dumbbell of 50% of the participant’s maximum shoulder elevation strength. Trial 3 was performed with a handheld dumbbell of 100% of the participant’s maximum shoulder elevation strength. Trial 4 was performed with a handheld dumbbell weight set to 125% of the participant’s maximum shoulder elevation strength. Each trial was performed one time per participant. For each trial the participant was instructed to elevate and lower their arm at a pace of three-seconds each through the full range of motion, with verbal pacing provided by the investigator. The participant was also advised to maintain full elbow extension, avoid trunk motion, and to reach full elevation ROM for each trial. If a participant failed to achieve any of these parameters during any trial as determined by visual inspection on Qualisys software, the trial was then repeated. Thirty seconds of rest was provided between each trial to minimize participant fatigue.

**Figure 1.**
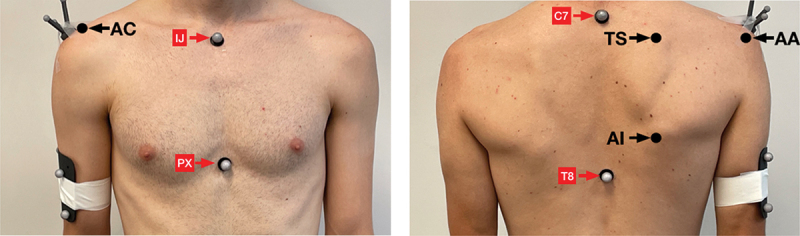
Kinematic Marker placement.

Note: Reflective markers are labeled in red. Digitized points are labeled in black. Incisura Jugularis (IJ), Processus Xiphoideus (PX), Acromioclaviculare (AC), 7th cervical spinous process (C7), 8th thoracic spinous process (T8), Trigonum Scapulae (TS), Angulus Acromialis (AA), Angulus Inferior (AI).

The clavicle, scapula, humerus, and trunk landmarks were tracked with 16 reflective markers affixed via 3 M Transpore Medical Tape and a Qualisys Camera System (Qualisys AB, Goteborg, Sweden) integrated with MotionMonitor software (Innovative Sports Training, Chicago, IL, USA). Both scapulae utilized a three non-collinear acromion marker cluster (AMC) placed on the spine of the scapula as it transitioned into the acromion. Bet-Or et al. (2017) found the AMC method to track scapula kinematics during shoulder elevation has high test–retest reliability (intraclass correlation coefficient = 0.90–0.98) and error of (SEM = max 2.3 deg, MDC = max 6.2 deg) for all 3 scapular rotations. Additional markers were placed on the 7th cervical vertebrae spinous process, 8th thoracic vertebrae spinous process, jugular notch, xiphoid process, a four-marker cluster on each arm midway between the lateral epicondyle and the lateral aspect of the acromion. Next, body landmarks were digitized of the AC joint (most dorsal point on the acromioclavicular joint), the angulus acromialis (most laterodorsal point of the scapula), the trigonum spinae scapula (midpoint of the triangular surface of the medial border of the scapula in line with the scapular spine), and the angulus inferior (most caudal point of the scapula). A local coordinate system, for each rigid segment, was created using a digitizing wand to digitize bony landmarks and each rigid segment was created while the subject was standing with arms at the side. Markers and digitizing points are shown in Figure 1. Euler angle sequences for humeral (Y – X’ – Y”), scapular (Y-X’-Z”), and clavicle (Y-X’-Z”) rotations were based on recommendations from the International Shoulder Group of the International Society of Biomechanics (Wu et al. 2005). In order to track scapular elevation via clavicular elevation, a vector between the angularis acromialis and the incisura jugularis was created. The angle of this vector relative to the transverse plane that bisects the incisura jugularis point represents scapular elevation. This measurement has been validated in previous literature (Karduna et al. 2001; McClure et al. 2001). Once the marker and digitization process were complete, participants performed the four shoulder elevation trials. Kinematic data of scapular upward rotation and clavicular upward elevation to define scapular elevation were extracted between 30 and 120 degrees of humeral elevation during humeral ascent and humeral descent. The amount (degrees) that the scapula moved within this range for both scapula elevation and upward rotation were recorded and are described in the results. 30 degrees of humeral elevation was selected as the starting range to have a consistent starting point among all subjects. 120 degrees was selected as the end range for consistency and validation studies have shown the acromion marker cluster as being a valid method for assessing scapula kinematics to this range of humero-thoracic elevation (Karduna et al. 2001; van Andel et al. 2009; Warner et al. 2012).

## Statistical analysis

Demographics and experimental measures were summarized using mean and standard deviation. Normality was assessed using Shapiro–Wilk test and box plots. A linear mixed effects multiple regression model (repeated measure) with a subject-level random intercept was used to examine the effect of load on the two scapular outcome variables (elevation and upward rotation) for the Control (unloaded) trial compared to each of the three loaded trials. A separate linear mixed effects multiple regression model with a subject-level random intercept was utilized to compare scapular elevation and upward rotation between the three loaded trials with each other (50% of 1 RM, 100% of 1 RM, and 125% of 1 RM). This multilevel modeling approach allows for multiple comparisons without adjusting the statistically significant level threshold (Yu et al. 2022). All tests were two-sided and conducted at a significant level of alpha = 0.05.

Data were analyzed using R software, (R Core Team Vienna, Austria).

## Results

**Figure 2.**
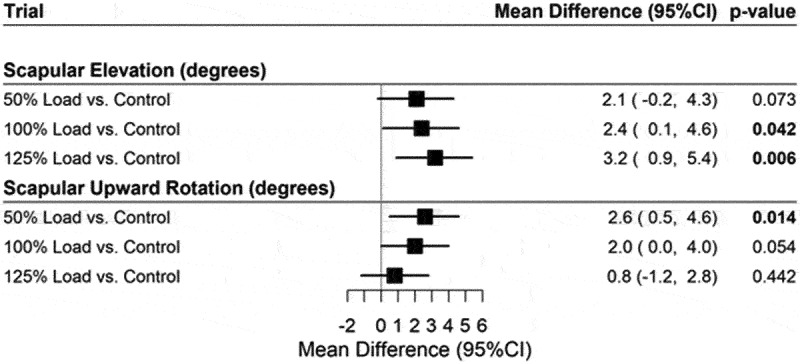
Plot for mean differences between loaded and control (unloaded) trials during humeral ascent.

Note: Adjusted for age, gender, and BMI.

Participant characteristics are shown in Table 1. During humeral ascent (Figure 2), a linear increase in scapular elevation is observed for increasing elevated loads with mean response = 2.1 degrees (deg) (95% CI: −0.2, 4.3), *p* = 0.073 for 50% Load vs Control, 2.4 deg (95% CI: 0.1, 4.6), *p* = 0.042 for 100% load vs control. 3.2 deg (95% CI: 0.9, 5.4), *p* = 0.006 for 125% load vs control respectively. In contrast, scapular upward rotation decreased with progressively higher load with mean response = 2.6 deg (95% CI: 0.5, 4.6), *p* = 0.014 for 50% load vs control. 2.0 deg (95% CI: 0.0, 4.0), *p* = 0.054 for 100% load vs control. 0.8 deg (95% CI: −1.2, 2.8), *p* = 0.442 for 125% load vs control, respectively.

**Table 1.** Participant characteristics (*n* = 50).

Variable	Mean ± SD
Age (years)	30.4 ± 8.5
Height (cm)	170.6 ± 10.5
Weight (kg)	71.8 ± 16.8
**Scapular Elevation (Deg)**	
Control	21.7 ± 8.2
50% Load	23.7 ± 8.4
100% Load	24.6 ± 8.8
125% Load	24.1 ± 8.2
**Scapular Upward Rotation (Deg)**	
Control	80.8 ± 10.4
50% Load	81.2 ± 9.6
100% Load	82.5 ± 9.2
125% Load	83.5 ± 9.0

**Figure 3.**
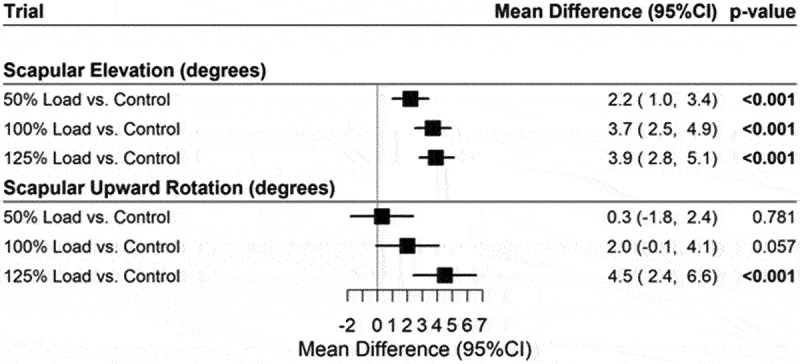
Forest plot for mean differences between loaded trials and unloaded (control) trials during humeral descent.

Note: Adjusted for age, gender, and BMI.

During humeral descent (Figure 3), scapular elevation increases with increasing elevated loads with mean response 2.2 deg (95% CI: 1.0, 3.4), *p* < 0.001 for 50% Load vs Control. 3.7 deg (95% CI: 2.5, 4.9), *p* < 0.001 for 100% load vs control. 3.9 deg (95% CI: 2.8, 5.1), *p* < 0.001 for 125% load vs control respectively. Similarly, scapular **upward rotation** increases with progressively higher load with mean response = 0.3 deg (95% CI: −1.8, 2.4), *p* = 0.781 for 50% load. 2.0 deg (95% CI: 0.0, 4.1), *p* = 0.057 for 100% load vs control. 4.5 deg (95% CI: 2.4, 6.6), *p* < 0.001 for 125% load, respectively.

**Figure 4.**
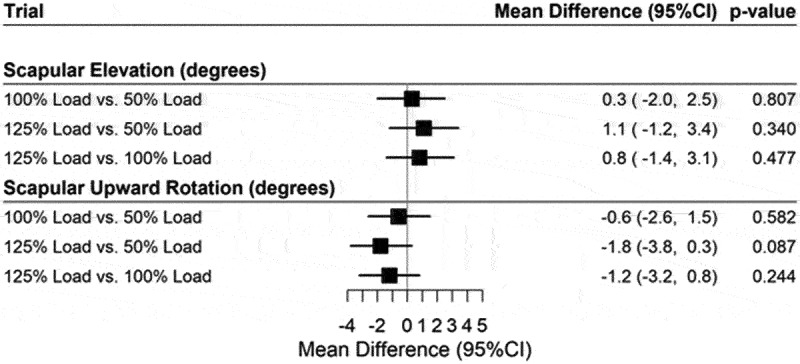
Forest plot for mean differences between loaded trials during humeral ascent.

Note: Adjusted for age, gender, and BMI.

The comparison of loaded trials during humeral ascent (Figure 4) results in means which are not statistically significant from each other for both scapular upward rotation and scapular elevation.

**Figure 5.**
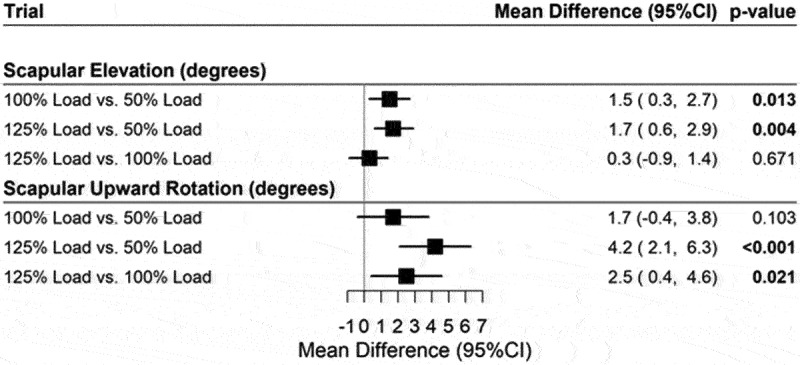
Forest plot for mean difference for all loaded trials during descent.

Note: Adjusted for age, gender, and BMI.

The comparison of loaded trials during humeral descent (Figure 5) shows significant differences between loaded trials for both scapular elevation and scapular upward rotation. For scapular elevation, the mean response = 1.5 deg (95% CI: 0.3, 2.7), *p* = 0.013 for 100% Load vs 50% load. 1.7 deg (95% CI: 0.6, 2.9), *p* = 0.004 for 125% load vs 50% load. 0.3 deg (95% CI: −0.9, 1.4), *p* = 0.671 for 125% load vs 100% load respectively. Similarly, for scapular upward rotation with mean response = 1.7 deg (95% CI: −0.4, 3.8), *p* = 0.103 for 100% load vs 50% load. 4.2 deg (95% CI: 2.1, 6.3), *p* < 0.001 for 125% load vs 50% load. 2.5 deg (95% CI: 0.4, 4.6), *p* = 0.021 for 125% load vs 100%, respectively.

## Discussion

This study investigated the effect of handheld external loading on scapular kinematics during shoulder elevation and lowering in healthy individuals. The results confirmed our hypothesis that scapular kinematics are altered with higher external loads. Specifically, scapular elevation increased during humeral ascent and descent, and scapular downward rotation increased during humeral descent. The scapular kinematic changes should be interpreted with caution, as the changes are small and most of the changes fall within the measurement error (MDC) of 2.7 degrees for scapula elevation and 5.0 degrees for scapular upward rotation (Michener et al. 2016).

While these findings emerge from a healthy population, the altered scapular kinematics when elevating with a higher load are consistent with the direction of altered kinematics seen in the shoulder pain population. The increased amount of scapular elevation, and increased amount of scapular downward rotation during descent seen with the higher external loads may imply that lifting with higher external loads may be a factor in the development of shoulder pain. Increased scapular elevation has been proposed as a risk factor for shoulder injuries in overhead athletes (Mell et al. 2005; Kibler et al. 2013). The current study demonstrated increased scapular elevation when the external load was increased by 25% higher than 1RM (Figure 2). Turgut et al. (2016) found increased scapular downward rotation in individuals with SAP. Within this study, there was no significant change in scapular upward rotation during humeral ascent with all external loads (Figure 2). However, during humeral descent the amplitude of scapular downward rotation was 4.5 degrees higher during the highest weighted trial as compared to the unweighted trial.

Identifying the amount of load that results in the compensatory scapular motion is relevant to inform load dose for strengthening exercises. This study demonstrated that during humeral descent there is a difference in both the amplitude of scapular elevation/depression and scapular upward/downward rotation when holding a weight at 125% of 1 RM as compared to holding a weight at 50% of the subjects 1 RM. Individuals that prescribe strengthening exercises and desire minimized alterations in scapular kinematics during humeral descent can elect to perform resistance exercises at 50% of the subjects 1 RM rather than 100% of the subjects 1 RM and above.

While an incrementally increased load did result in some kinematic change during humeral ascent, the degree of kinematic change is more significant during humeral descent where shoulder girdle muscles primarily function eccentrically. This finding emphasizes the need to assess scapular kinematics during humeral descent. Altered scapular kinematics during humeral descent, especially a greater amplitude of movement into downward rotation may ultimately place more stress on the RC tendons than the stress placed on the tendons during humeral ascent.

There are some study limitations worth noting. First, the altered kinematics with higher loads are noticed when an individual lifts at 50%, 100% and 125% of their 1 RM, but typically athletes perform repetitive overhead tasks rather than a single repetition per trial. This was done to minimize the effect of fatigue, but it prevents the more detailed analysis that multiple repetitions would allow. Second, it is possible that individuals experience altered scapular kinematics when lifting at lower than 50% of their 1 RM as well. Additional study limitations include that the study did not randomize the order of trials potentially resulting in altered scapular kinematics due to the order of trials. Performing the trials in a non-randomized manner could result in changes associated with fatigue rather than increases in the external load. Controlling for findings associated with fatigue was attempted via providing ample rest time between trials (30 s), however fatigue may have played a role in the results. Future studies should investigate the effect of fatigue on scapular kinematics. Finally, it is important to exercise caution when extrapolating our findings to individuals experiencing shoulder pain, as the subjects in our study consisted of healthy individuals who were free from pain. Future studies should also investigate the effect external loads have on scapular kinematics in a painful population. Comparing the findings from healthy controls with individuals who have shoulder pain may assist in identifying the scapular kinematics that prevent injury as well as those that contribute to injuries.

This study showed increasing scapular elevation during a shoulder elevation task with increasing external load. Further, the study found a greater amplitude of movement in the direction of downward rotation during descent. Those who wish to minimize altered scapular kinematics during overhead strength training to minimize injury risk can elect to monitor the prescribed load. Although this study investigated healthy controls, these findings may support a framework to optimize scapular kinematics in a group with shoulder pain. That framework likely needs a comparison study which investigates whether external loads affect scapular kinematics in a painful population.

## Conclusion

The study investigated the effect of external loading on scapular kinematics during shoulder elevation and lowering in healthy individuals. The results showed that an external load above 1RM increased scapular elevation during humeral ascent, but there was no significant change in scapular upward rotation. During humeral descent, the amplitude of scapular elevation/depression and scapular upward/downward rotation during weighted trials was higher than the unweighted trial.
