# Correction

**DOI:** 10.1080/23335432.2025.2569220

**Published:** 2025-10-10

**Article title**: External handheld loads affect scapular elevation and upward rotation during shoulder elevation tasks

**Authors**: Alan Eldridge, Everett Lohman, Skulpan Asavasopon, Lida Gharibvand, & Lori Michener

**Journal**: *International Biomechanics*

**Bibliometrics**: Volume 11, Number 01, pages 12-19

**DOI**: https://doi.org/10.1080/23335432.2024.2332212

When this article was first published, there was an error in the pagination of the article. This has now been corrected online.

Taylor & Francis apologizes for this error.

